# The Treatment of Insanity: Existing Defects and their Remedy

**Published:** 1907-05-18

**Authors:** 


### THE TREATMENT OF INSANITY.

### Existing Defects and their Remedy.

### VI. SCIENTIFIC METHODS IMPOSSIBLE UNDER ASYLUM SYSTEM.

From what has been said it will appear that no sensible improvement in the treatment of insanity is to be expected from the public asylums of this country. Scientific investigation is outside of their province; they have neither the will, nor the means, nor the men to devote to it. With the registered hospitals the case is otherwise. These are in their foundations much on a par with the charitable general hospitals. They are endowed; they are supported to some extent by voluntary subscriptions, and as they make large profits from the payments of the patients, they have very considerable sums at their command which they must sometimes find it difficult to spend. Some of this money goes, as we have seen, to extending the size and increasing the luxury of the institution; some of it goes in relief of the fees of poor patients; some of it goes in increased salaries and a larger number of officials; and some of it is spent in a manner which shows clearly how difficult it is to find objects for the expenditure. Costly chapels, theatres, concert-halls, laundries, and other buildings absorb many tens of thousands of pounds. Antique furniture of choice and costly description and other unnecessary luxuries indicate the difficulty that is felt in disposing of these funds. If a portion of the money thus injudiciously lavished had been spent on the provision of laboratories; if the additions to the staff had taken the form of skilled and zealous workers at research, the reproach of alienism might long ago have been removed, and there would have been no need for these articles. The Royal Hospital of Bethlem is in an eminently favourable situation for such an equipment. It receives none but recent and presumably curable cases, the very cases to which treatment can be most favourably applied. It is in the middle of the greatest city of the world, surrounded by many medical schools, great and small, from amongst which it might have the pick of the best men, all eager to find a field of discovery, hitherto uncultivated, to which they can devote their energies. It is not, it is true, one of the wealthy hospitals for the insane, but if the will and the enterprise had been there, it might occupy its natural position, of the foremost school of alienism in the world. One would have supposed that if it had not the funds, and it is difficult to believe that the funds could not have been found, to form an equipment of its own, it might have made an arrangement with one of the general hospitals to get the work done. But it has done nothing, and it is no longer to be expected that it will do anything.

It appears that it is hopeless to expect this great task to be taken up by any of the existing institutions. For one reason or another they are unfitted or unwilling to undertake it. The efforts that have been made have been so far but tentative and partial. Buildings have been provided, but have not been put to the use that was intended; laboratories have been equipped, but have not been supported by adequate clinical study. Something more must be done, and must be done in a new way and by new men. The "hospital treatment" of the insane meets with a certain amount of ridicule from experienced alienists; and if, as some of both supporters and opponents of the plan seem to infer, "hospital treatment" means keeping patients in bed and recording their physical signs, the ridicule is justified. Alienists of much experience and high character meet the demand in some such terms as these: "You say we do not treat our patients; and it is true that we do not apply poultices to their heads or blisters to their feet; we do not continually dose them with the last new coal-tar derivative; we see no prospect of benefit from the Röntgen rays or the Finsen light; but we attend to the general health; we build up the nutrition; we treat any bodily disease there may be; we are careful to procure them sufficient sleep; we place them in circumstances in which they are protected from the stress and worry and responsibility of ordinary life; and what more can we do? We cannot give a melancholy person two tablespoonfuls of happiness three times a day out of a bottle. What do you suggest that we can do more? What would you, with your hospital and your laboratories and your army of clinical observers, do that we do not do now?"

The answer is: That is what we have to find out. We have to discover what it is that is causing the insanity in a given case, and then set ourselves to counteract that cause. It is certain, for instance, that some cases of insanity are produced by the action of poisons circulating in the blood. We can actually produce insanity by administering a poison; we can vary the depth of the insanity by varying the amount and the rate of the administration of the poison; and we can cure the insanity by withdrawing the poison. Everyone who has observed a case of drunkenness is witness to the truth of this statement. But acute insanity more or less resembling drunkenness is a very frequent form of insanity. It is one of the most frequent forms of the recurring forms of insanity. Is it not likely, then, that in very many cases acute insanity is due to the action of some poison or other, it may be the same in some or many cases? And is it not fair to suppose that with sufficient means at our disposal we might be able to discover the nature and origin of this poison in at least some cases, and to find the means of neutralising it? How many cases that end in utter mental wreck—or in what is almost worse, permanent distressing delusion—might not be cut short in an early stage, and restored to complete sanity before any permanent damage to the brain has been done? If it would save even a minority of those who slide every year from acute into permanent insanity from becoming for the rest of their lives burdens on the community, and retain them in the wealth-producing class, would it not pay?

We have put but one case, the case of acute insanity due to poison; but there are others. There is the fell disease, general paralysis of the insane. Owing to the splendid work of Dr. Mott in the London County Laboratory, we now know that syphilis is a main factor, if indeed it is not the main factor, in the production of this disease. That it is due to the action of a poison in some way derived from the syphilitic virus there is now no doubt, but the knowledge that we have gained serves to show more clearly the vast extent of the knowledge that we lack. How is the poison derived from the syphilitic virus? What relation does the one bear to the other? In what direction are we to look for an antidote? For an antidote there certainly is, waiting to be discovered. Of sufferers from this disease 1,330 are admitted to the asylums of England every year. Remember that when a remedy for general paralysis is discovered, at the same moment is discovered a remedy for tabes. Is it not worth while to try?

